# Regaining the senses of touch and movement

**DOI:** 10.7554/eLife.36137

**Published:** 2018-04-10

**Authors:** Victor de Lafuente

**Affiliations:** Institute of NeurobiologyNational Autonomous University of MexicoQuerétaroMéxico

**Keywords:** intracortical microstimulation, somatosensory cortex, brain-machine interface, proprioception, somatosensation, paraplegia, Human

## Abstract

Artificially activating neurons in the cortex can make a tetraplegic patient feel naturalistic sensations of skin pressure and arm movement.

**Related research article** Salas MA, Bashford L, Kellis S, Jafari M, Jo H, Kramer D, Shanfield K, Pejsa K, Lee B, Liu C, Andersen RA. 2018. Proprioceptive and cutaneous sensations in humans elicited by intracortical microstimulation. *eLife* **7**:e32904. doi: 10.7554/eLife.32904

When we reach for a cup of coffee, our muscles and joints send feedback signals that inform our brain about changes in the position of our limbs and the forces acting on them. Then, when we actually grab the cup, our skin helps us make a successful grip by sensing that we have made contact with an object and relaying information about its temperature, weight and so on (Johansson and Flanagan, 2009). Without proprioceptive information – the sensory feedback from our tendons and muscles – and our sense of touch, it would be extremely difficult to perform even trivial tasks.

This is especially relevant to efforts to help people with damaged limbs or spinal cords regain their independence (Nicolelis, 2003). Thanks to recent advances in neuroscience and engineering, such patients can now be equipped with sophisticated robotic prostheses that enable them to walk or reach for objects. But these individuals are still missing the important senses of touch and proprioception that are needed to feel and control either paralyzed or artificial limbs (Bensmaia and Miller, 2014).

Now, in eLife, Richard Andersen of the California Institute of Technology and colleagues – including Michelle Salas and Luke Bashford as joint first authors – report that it is possible, in principle, to restore lost tactile and proprioceptive sensations in humans by directly activating the relevant neurons in the brain (Salas et al., 2018). In an amazing feat of neurosurgery and engineering, a tetraplegic patient was surgically implanted with metal electrodes in the somatosensory cortex, the area of the brain that processes internal and external sensations. Small electric currents were then injected through these electrodes, activating the neurons in the vicinity.

The power of intracortical microstimulation, as this technique is called, was first demonstrated almost 30 years ago when William T. Newsome and colleagues used it to show that perception of visual motion could be manipulated by injecting small currents near motion-sensitive neurons in the cortex of Rhesus monkeys (Salzman et al., 1990). As they wrote in their original report: “… it is remarkable that local microstimulation of directionally selective neurons can cause a substantial change in perception.” More recent work, also in non-human primates, has revealed that artificially activating touch or vision-related neurons elicits behavioral responses that are consistent with the animals having felt a mechanical vibration or a visual stimulus (Romo et al., 1998; Murphey and Maunsell, 2007).

However, microstimulation experiments in non-human animals cannot provide us with information about the subjective quality of the sensations evoked by the artificial activation of neurons. What does having information directly fed to our cortical neurons actually feel like? This question can only be answered by performing experiments on humans. A recent groundbreaking experiment by Robert Gaunt and collaborators, in which they used microstimulation on a tetraplegic patient, revealed that the majority of the tactile sensations evoked by the technique felt possibly natural by the participant (Flesher et al., 2016).

The new study by Salas et al. – who are based at Caltech, the Keck School of Medicine of USC and the Rancho Los Amigos National Rehabilitation Center – goes a step further by being able to generate sensations of proprioception. Salas et al. showed that microstimulation of touch-related neurons within the somatosensory cortex of a tetraplegic patient can evoke natural sensations, similar to those experienced before the injury. In particular, the patient reported feeling sensations that resembled skin pressure, tapping and vibration, and also proprioceptive experiences that are normally associated with movements of the arm and hand. Microstimulation therefore appears to be a promising therapeutic way to restore both touch and proprioception in tetraplegic individuals.

However, for the technique to be an effective therapy, the interface between the electrode array and the cortical neurons needs to remain viable for years, if not decades. For this to be possible, future research should focus on replacing metal electrodes with electrodes made from new materials that are better suited to providing a long-lasting neuronal interface to the brain (Bareket-Keren and Hanein, 2012). In turn, this interface would allow the plastic nature of the brain to learn, better interpret, and ultimately embody the artificial electric signals originating from the neuronal implant. More generally, a successful neural interface would permit a constant and seamless interaction between our brains and future advances in technology. In addition to helping patients recover lost functions, such an interface might also help them gain new abilities like enhanced vision and hearing, or perception of magnetic fields.
